# Supplementary material for: A fault-intrusion-tolerant system and deadline-aware algorithm for scheduling scientific workflow in the cloud
Source: PeerJ Comput Sci. 2021 Nov 2;7:e747. doi: 10.7717/peerj-cs.747 (PMC8576554; doi:10.7717/peerj-cs.747)
Supplement: Supplemental Information 6 [file peerj-cs-07-747-s006.zip › Myproject1.1/docs/org/workflowsim/class-use/Job.html]

Uses of Class org.workflowsim.Job


JavaScript is disabled on your browser.


- Overview
- Package
- Class
- Use
- Tree
- Deprecated
- Index
- Help

- Prev
- Next

- Frames
- No Frames

- All Classes

## Uses of Class org.workflowsim.Job

- Packages that use Job

  | Package | Description |
  | --- | --- |
  |  |  |
  | --- | --- |
  | org.workflowsim |  |
  | org.workflowsim.clustering |  |
  | org.workflowsim.failure |  |
  | org.workflowsim.reclustering |  |
  | org.workflowsim.utils |  |
- - ### Uses of Job in org.workflowsim

    Fields in org.workflowsim with type parameters of type Job

    | Modifier and Type | Field and Description |
    | --- | --- |
    |  |  |
    | --- | --- |
    | `protected java.util.List<Job>` | ClusteringEngine.`jobList` The job list |

    Methods in org.workflowsim that return types with arguments of type Job

    | Modifier and Type | Method and Description |
    | --- | --- |
    |  |  |
    | --- | --- |
    | `java.util.List<Job>` | ClusteringEngine.`getJobList()` Gets the job list. |

    Method parameters in org.workflowsim with type arguments of type Job

    | Modifier and Type | Method and Description |
    | --- | --- |
    |  |  |
    | --- | --- |
    | `protected void` | ClusteringEngine.`setJobList(java.util.List<Job> jobList)` Sets the job list. |
  - ### Uses of Job in org.workflowsim.clustering

    Methods in org.workflowsim.clustering that return Job

    | Modifier and Type | Method and Description |
    | --- | --- |
    |  |  |
    | --- | --- |
    | `protected Job` | BasicClustering.`addTasks2Job(java.util.List taskList)` Add a list of task to a new job |
    | `protected Job` | BasicClustering.`addTasks2Job(Task task)` Add a task to a new job |

    Methods in org.workflowsim.clustering that return types with arguments of type Job

    | Modifier and Type | Method and Description |
    | --- | --- |
    |  |  |
    | --- | --- |
    | `java.util.List<Job>` | BasicClustering.`getJobList()` Gets the job list |
    | `java.util.List<Job>` | ClusteringInterface.`getJobList()` get job list. |
  - ### Uses of Job in org.workflowsim.failure

    Methods in org.workflowsim.failure with parameters of type Job

    | Modifier and Type | Method and Description |
    | --- | --- |
    |  |  |
    | --- | --- |
    | `static boolean` | FailureGenerator.`generate(Job job)` Generates a failure or not |
  - ### Uses of Job in org.workflowsim.reclustering

    Methods in org.workflowsim.reclustering that return types with arguments of type Job

    | Modifier and Type | Method and Description |
    | --- | --- |
    |  |  |
    | --- | --- |
    | `static java.util.List<Job>` | ReclusteringEngine.`process(Job job, int id)` Process job recreation based on different reclustering algorithm |

    Methods in org.workflowsim.reclustering with parameters of type Job

    | Modifier and Type | Method and Description |
    | --- | --- |
    |  |  |
    | --- | --- |
    | `static java.util.List<Job>` | ReclusteringEngine.`process(Job job, int id)` Process job recreation based on different reclustering algorithm |
  - ### Uses of Job in org.workflowsim.utils

    Methods in org.workflowsim.utils with parameters of type Job

    | Modifier and Type | Method and Description |
    | --- | --- |
    |  |  |
    | --- | --- |
    | `double` | OverheadParameters.`getPostDelay(Job job)` Gets the postscript delay for a particular job based on the depth(level) |

- Overview
- Package
- Class
- Use
- Tree
- Deprecated
- Index
- Help

- Prev
- Next

- Frames
- No Frames

- All Classes
